# Anti-metastatic and anti-proliferative activity of eugenol against triple negative and HER2 positive breast cancer cells

**DOI:** 10.1186/s12906-018-2392-5

**Published:** 2018-12-05

**Authors:** Mashan L. Abdullah, Mohamed M. Hafez, Ali Al-Hoshani, Othman Al-Shabanah

**Affiliations:** 1 Pharmacology and toxicology department, King Saud University, Riyadh, Saudi Arabia; 2 Experimental Medicine Department-MNGHA, King Abdullah International Medical Research Center/King Saud bin Abdulaziz University for Health Sciences, Riyadh, 11426 Saudi Arabia; 3 Cancer Biology Department,Virology and Immunology Unit, National Cancer Institute, Cairo University, Cairo, 11976 Egypt

**Keywords:** Eugenol, Anti-proliferative, Anti-apoptotic, Gene expression, Triple negative breast cancer

## Abstract

**Background:**

Eugenol is a natural phenolic compound and possesses anticancer and antibacterial activities. Breast cancer is a major global health problem, and most of the chemotherapeutic agents are highly toxic with long-term side effects. Therefore, this study aimed to explore the possibility of using eugenol as an anti-metastatic and anti-proliferative agent against MDA-MB-231 and SK-BR-3 breast cancer cells.

**Methods:**

Breast cancer cell lines MDA-MB-231 and SK-BR-3 were treated with eugenol and cell proliferation was measured using a real-time cell electronic sensing system. Annexin V analysis with flow cytometry was used to detect the effect of eugenol on cell death. In MDA-MB-231 and SK-BR-3 cells, metastatic potential after eugenol treatment was examined using a wound-healing assay. Real-time PCR was used to study the effect of eugenol on the expression of anti-metastatic genes such as *MMP2, MMP9,* and *TIMP-1,* and genes involved in apoptosis including *Caspase3, Caspase7*, and *Caspase9*.

**Results:**

Treatment with 4 μM and 8 μM eugenol for 48 h significantly inhibited cell proliferation of MDA-MB-231, with an inhibition rate of 76.4%, whereas 5 μM and 10 μM of eugenol for 48 h significantly inhibited the proliferation of SK-BR-3 cells with an inhibition rate of 68.1%. Eugenol-treated cells showed significantly decreased *MMP2* and *MMP9* expression and an insignificant increase in *TIMP1* expression in HER2 positive and triple negative breast cancer cells. Eugenol significantly increased the proportion of MDA-MB-231 and SK-BR-3 cells in late apoptosis and increased the expression of *Caspase3, Caspase7,* and *Caspase9*.

**Conclusion:**

To the best of our knowledge, this is the first study to describe the anti-metastatic effect of eugenol against MDA-MB-231 and SK-BR-3 breast cancer cell lines.

## Background

Breast cancer is a major health problem with high morbidity and mortality rates among women worldwide [1]. Its high prevalence and differences in response patterns to various treatment modalities and clinical outcomes provides a strong rationale for identifying natural and synthetic cancer preventive agents [2]. Breast cancer is a heterogeneous complex of diseases [3] and can be classified into different subtypes based on biological characteristics (traditional classification systems), or gene expression patterns (molecular classification) [4]. Triple-negative breast cancer (TNBC) has a distinct aggressive molecular subtype, characterized by lack of progesterone receptor (PR), estrogen receptor (ER), and human epidermal growth factor receptor-2 (HER-2) expression [5] resulting in poorer outcomes than other subtypes [6]. Moreover, TNBC can be classified in different subtyping based on gene expression profiling into basal like1, basal-like 2, an immunomodulatory subtype, mesenchymal, mesenchymal stem cell-like and luminal androgen receptor subtype [7]. Because of the absence of commonly targeted receptors present in other breast tumor subtypes, agents that specifically target TNBC are not yet available. Another type of breast cancer that has aggressive biological behavior is *HER2-* positive breast cancer which categorized by high HER2 expression [3].

In breast cancer patients, metastasis is considered one of the main causes of death [8]. Metastasis starts with degradation of the extracellular matrix, followed by cell invasion and trans-endothelial cell migration and ends with colonization in new site [9]. In metastasis, there was a link between the high levels of a group of matrix metalloproteinases (MMPs), a family of 23 structurally and functionally related endopeptidases [10], and most human tumor cell lines [11]. During tumor progression, the MMPs produce extracellular matrix remodeling and release of cytokines and growth factors that causes modification for the microenvironment [12]. Several MMPs (like MMP-1, − 2, − 3, − 7, − 9, − 11 and − 14) have different roles in different cancer stages [13, 14]. The MMP-2 and -9 are involved in tumor angiogenesis mostly via their matrix-degrading capacity and neovascularization potential [15]. In breast cancer patients, the level MMP-2 and MMP-9 are overexpressed [13] which is associated with a shortened relapse-free survival [16].

Matrix metalloproteinases activities and function were regulated by the tissue inhibitor of metalloproteinase (TIMP) family which includes four subtypes (TIMP-1, 2, 3, and 4). Down-regulation of TIMPS shows some apoptotic properties in different cancer cell lines [17]. TIMP-3 overexpression is associated with apoptosis in lung cancer cell lines. The TIMPs overexpression can reduce the metastasis of cancer [18], for example, TIMP1 overexpression slows the carcinogenesis process in transgenic mice [19], whereas, TIMP-2 is involved in carcinogenesis and metastasis, and is downregulated in prostate cells and tumor samples [20].

A large number of natural products have chemo-preventive potential with no side effects [21]**.** Eugenol is listed by the Food and Drug Administration as “Generally Regarded as Safe” when consumed orally in the unburned form [22]. Eugenol is a natural phenolic compound available in honey and the essential oils of cloves, cinnamon, and other aromatic spices. It is added as a therapeutic ingredient in various medications to treat digestive disorders [23] and as an antiseptic, analgesic [24], anti-inflammatory, antimicrobial [25] and antioxidant agent [26]. Furthermore, eugenol has several anticancer properties in colon, liver, prostate, and breast cancer [22, 27]. Eugenol prevents cancer progression by modulating the expression of several genes involved in cell growth, angiogenesis, and apoptosis [22]. Moreover, in a rat model of gastric carcinogenesis, eugenol was observed to induce apoptosis and inhibit invasion and angiogenesis [28].

Up to date, we could not find any study in the literature, describing the anti-metastatic activity of eugenol against triple negative (MDA-MB-231) and anti-metastatic, anti-proliferative and apoptotic activity of eugenol against HER2 positive (SK-BR-3) breast cancer cells. Therefore, this study aimed to assess the effect of eugenol on the proliferation, metastasis, and apoptosis of triple-negative MDA-MB-231 and HER2-positive SK-BR-3 breast cancer cell lines.

## Methods

### Reagents

Eugenol and Trypan blue solution were purchased from Sigma Aldrich (Sigma Aldrich, USA). TaqMan probes, Gene expression PCR Master Mix kit, and High Capacity cDNA Reverse Transcription kit were purchased from Applied Biosystems (Life Technologies, Grand Island, NY, USA). MDA-MB-231 (ATCC HTB-26®) and SK-BR-3 (ATCC HTB-30™) cells were obtained from American Type Culture Collection (Rockville, MD, USA). Dulbecco’s Modified Eagle’s Medium (DMEM), Roswell Park Memorial Institute (RPMI) medium, TRIzol reagent, and Muse™ Annexin V & Dead Cell Kit were purchased from Merck KGaA© (Darmstadt, Germany). MTT reagent was purchased from Roche (Roche Diagnostics, Mannheim, Germany). Western blot detection kits, Luminata® Western HRP Chemiluminescence Substrates were purchased from EMD Millipore (Billerica, MA).

### Cell viability assay using MTT

Viability of triple negative- (MDA-MB-231) and HER2 positive- (SK-BR-3) breast cancer cells in response to the different concentrations of eugenol was determined by measuring the capacity of cellular oxidoreductase enzymes present in viable cells to convert the tetrazolium dye 3-(4,5-dimethylthiazol-2-yl)-2,5-diphenyl tetrazolium bromide (MTT) to its insoluble formazan form. In brief, cells were seeded in 96-well plates (1 × 10^4^ cells/well) and incubated for 24 h. The medium was then replaced with fresh medium containing different concentrations of eugenol; untreated cells were treated with Dimethyl sulfoxide (DMSO). At the end of 24 h, 10 μl of MTT reagent was added to each well, and the plates were incubated for 4 h at 37 °C. Next, 100 μl of isopropanol was added to each well, and after 15 min, the amount of formazan was quantified by measuring absorbance at 450 nm using an ELISA reader. Percentage of cell proliferation was calculated relative to control wells designated as 100% viable cells, where % cell proliferation = (A treated)/(A control) × 100.

### Cell proliferation analysis using real-time cell electronic sensing system

MDA-MB-231 and SK-BR-3 were cultured individually in 100 μl complete medium containing 2 × 10^4^ cells in each well of 16-well microtiter Eplates with integrated microelectronic sensor arrays at the bottom of each well. The plates were incubated for 30 min, then inserted into a Real-Time Cell Electronic Sensing System (ACEA Biosciences Inc., San Diego, CA). The eugenol was added to each well after the cell growth reaches the log phase, then the plates were inserted into the Real-Time Cell Electronic Sensing System, finally were incubated for 72 h. (for label-free and dynamic monitoring of cell proliferation). The electronic readout, cell-sensor impedance, is displayed as arbitrary units called the cell index.

### RNA extraction and cDNA synthesis

TRIzol reagent (Invitrogen®) was applied to isolate total RNA according to the standard protocol as described previously [29]. The quality and quantity of isolated RNA were assessed by measuring absorbance at 260 nm and maintaining a 260/280 ratio of ~ 2.0. First strand cDNA was synthesized from 1 μg total RNA using the High-Capacity cDNA reverse transcription kit (Applied Biosystems®) according to the manufacturer’s instructions as described previously [30].

### Real-time polymerase chain reaction

To determine the effect of eugenol on gene expression levels, quantitative real-time polymerase chain reaction (qRT-PCR) was conducted. MDA-MB-231 cells were treated with 4 μM and 8 μM eugenol whereas SK-BR-3 was treated with 5 μM and 10 μM eugenol for 48 h. These doses and times were chosen based on the cytotoxicity results. Quantitative PCR was performed to detect the expression levels of *MMP2* (Hs01548724_m1), *MMP9* (Hs00957562_m1), and *TIMP1* (Hs01092511_m1) using the gene expression master mix (Applied Biosystems, CA, USA), on the ABI PRISM 7500 Detection System (Applied Biosystems, USA). Glyceraldehyde-3-phosphate dehydrogenase (Hs02786624_g1) was used as an internal control for qRT-PCR. Each sample was analyzed in triplicate, and representative data sets are shown. Results were analyzed using the 2^−ΔΔCT^ method. Data were expressed as mean fold changes ± standard deviation (SD) for three independent amplifications.

### Western blot analysis

Total protein was extracted from cells after treatment with eugenol, and protein concentrations were determined using NanoDrop (NanoDrop 8000, Thermo Scientific, USA). Western blot was performed according to our previous study [31] using appropriate primary antibodies (Cell Signaling, Danvers, MA, USA): Total and cleaved caspase-3 (9662), Total and cleaved caspase-9 (9508), Caspase-7 (D2Q3L), MMP-9 (D6O3H) XP, MMP-2 (D4M2N), TIMP1 (D10E6), GAPDH (D16H11) XP, followed by HRP-linked secondary Antibody (7074) or (7076). Then the Bands were visualized using the Documentation System; images were captured and analyzed using Quantity One software, (Bio-Rad, USA). The relative quantification was calculated by dividing the density of the target protein with the respective loading control. GAPDH was used as the loading control.

### Flow cytometric analysis of apoptosis

The percentage of cells undergoing apoptosis was determined by flow cytometry using annexin V staining. According to the manufacturer’s instructions, MDA-MB-231 and SK-BR-3 cells were treated with different doses of eugenol for 48 h, the medium was removed, and cells were washed with cold PBS. Cells were collected after trypsinization by centrifugation at 300×*g* for 5 min and then resuspended in 0.5 ml PBS. Further, 100 μl of cell suspension was incubated with 100 μl Muse™ Annexin V & Dead Cell reagent in the dark for 20 min at 25 °C and immediately analyzed on the Muse™ Cell Analyzer (Merck KGaA Co., Darmstadt, Germany) to determine the viable, apoptotic, and necrotic cells populations.

### Wound healing assay

Migration potential of MDA-MB-231 and SK-BR-3 cells after eugenol treatment was determined using a wound healing assay. MDA-MB-231 and SK-BR-3 cells (5 × 10^5^) were cultured in 6-well plates and grown to 70–80% confluence. Subsequently, the cells were scratched using a 200-μl pipette tip when they grew to 90% confluence. The cells were washed twice using PBS, and the specific medium for each cell was completely replaced. Different concentrations of eugenol were added to the plate and incubated for 48 h. Wound closure was recorded using an Olympus inverted microscope (Olympus, Tokyo, Japan). To evaluate cell migration ability, at least 5 random fields were captured for each time point at 0, 24, and 48 h. The distance migrated by the cell monolayer to close the wounded area during this period was measured and analyzed using Image J software. Experiments were performed in triplicate and repeated at least three times.

### Statistical analyses

Comparative analysis of results from various experimental groups with their corresponding controls was performed using Graph Pad 5.0 Prism software (GraphPad Software, Inc., La Jolla, CA, USA). Data were stated as mean ± standard deviation (SD) (from triplicate experiments). One-way analysis of variance (ANOVA) followed by Tukey–Kramer post-ANOVA test was used to measure the treated groups. Differences were considered significant when *p <* 0.05.

## Results

### Effect of eugenol on cell viability

**Fig. 1:**
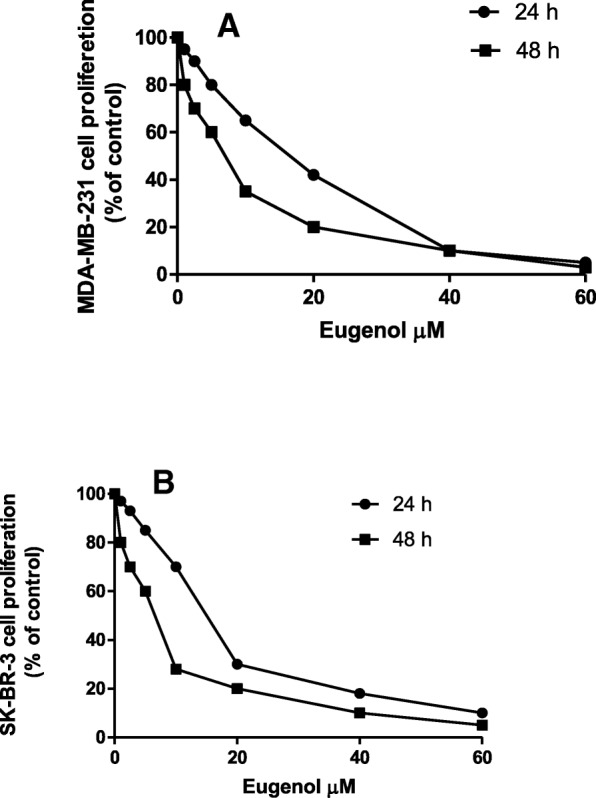
Effect of eugenol on MDA-MB-231 (**a**) and SK-BR-3 (**b**) cell proliferation. MDA-MB-231 and SK-BR-3 cells were treated with different concentrations of eugenol, and cell viability was determined using the MTT assay. Values represent % of the control

To determine the anti-proliferative effect of different eugenol concentrations against MDA-MB-231 and SK-BR-3 cell growth, the cell proliferation assay was performed using the MTT method and real-time cell analysis. Figure 1 shows that eugenol at concentrations up to 2.5 μM did not significantly affect cell viability. However, concentrations of 5, 10, and 20 μM significantly decreased the viability of MDA-MB-231 cells by approximately 20, 35, and 58%, respectively, at 24-h incubation period, whereas, at 48-h incubation with eugenol 5, 10, and 20 μM significantly reduced the cell viability by approximately 40, 65, and 80%, respectively. On the other hand, high eugenol concentrations (40 and 60 μM) markedly inhibit cell proliferation by > 90%. Eugenol at concentrations 5, 10, and 20 μM significantly decreased SK-BR-3 cell viability by approximately 15, 30, and 70% respectively upon 24 h incubation, whereas 48 h incubation with eugenol at 5, 10, and 20 μM reduced the cell viability by approximately 32, 72, and 80%, respectively. The data obtained data from MTT, and real-time cell analysis showed almost the same results of cell viability.

### Effect of eugenol on cell growth and proliferation

**Fig. 2:**
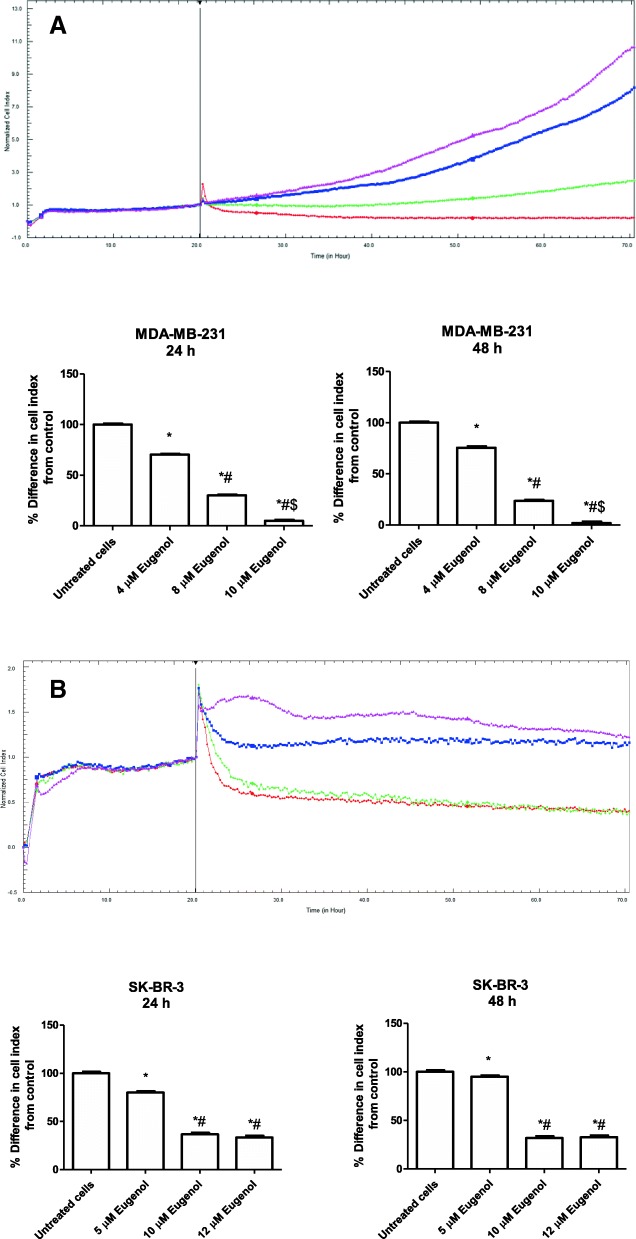
**a** Proliferation curve of MDA-MB-231 cells obtained by real-time analysis. Cells were seeded in E-16 Plates at 2 × 10^4^ cells/well and were continuously observed by measuring the cell index (CI) values for 72 h. Cell proliferation was observed at intervals of 15 min. The black line marks the normalization of the CI time point at 20 h. MDA-MB-231 cells were then treated with different doses of eugenol. Colored curves represent the various concentrations of eugenol. Pink line: Control (DMSO); Blue line: 4 μM Eugenol; Green line: 8 μM Eugenol; Red line: 10 μM Eugenol. Data are presented as mean ± SD (*n* = 3). *, # and $ indicate a significant change from untreated cells, 4, and 8 μM eugenol, respectively, at *p* < 0.05 using ANOVA followed by Tukey–Kramer post-ANOVA test. **b** Proliferation curve of SK-BR-3 cells obtained by real-time analysis. Cells were seeded in E-16 Plates at 2 × 10^4^ cells/well and were continuously observed by measuring CI values for 72 h. Cell proliferation was observed at intervals of 15 min. The black line marks the normalization of the CI time point at 20 h. SK-BR-3 cells were then treated with different doses of eugenol. Colored curves represent various concentrations of eugenol. Pink line: Control (DMSO); Blue line: 5 μM Eugenol; Green line: 10 μM Eugenol; Red line: 12 μM Eugenol. Data are presented as mean ± SD (*n* = 3). * and # indicate significant changes from untreated and 5 μM eugenol, respectively, at *p* < 0.05 using ANOVA followed by Tukey–Kramer post-ANOVA test

The effect of eugenol concentrations on MDA-MB-231 and SK-BR-3 cell proliferation was also investigated using real-time cell analysis. Results indicated that eugenol could regulate the cell proliferation of MDA-MB-231 and SK-BR-3 cells (Fig. 2). Figure 2a shows that incubation of MDA-MB-231 for 24 and 48 h with 4 μM eugenol significantly inhibits MDA-MB-231 cell proliferation by 29.7 and 24.5% respectively (*p* < 0.05) compared to untreated cells, and incubation with 8 μM eugenol for the same period results in significantly reduced cell proliferation by 70 and 76.4% respectively compared to that of untreated cells (*p* < 0.05). Eugenol 10 μM could thus be a toxic dose wherein incubation with 10 μM eugenol for 24 and 48 h significantly decreased MDA-MB-231 cell proliferation by 95.1 and 97.1%, respectively compared to untreated cells (*p* < 0.001). Similarly, incubation with 5 μM eugenol for 24 and 48 h significantly reduced SK-BR-3 proliferation by 20 and 5% respectively (*p* < 0.05). Eugenol at 10 μM significantly decreased SK-BR-3 proliferation by 63.3 and 68.1% at 24 and 48 h, respectively, compared to untreated cells (*p* < 0.001). Similarly, incubation with 12 μM eugenol for 24 and 48 h caused a significant decrease in SK-BR-3 cell proliferation by 66.7 and 67.3%, respectively, compared to untreated cells (Fig. 2b). Based on these findings, eugenol at 4 and 8 μM for MDA-MB-231 and 5 and 10 μM for SK-BR-3 at the 48 h time interval were used in all subsequent experiments.

### Effect of different concentrations of eugenol on cell migration

**Fig. 3:**
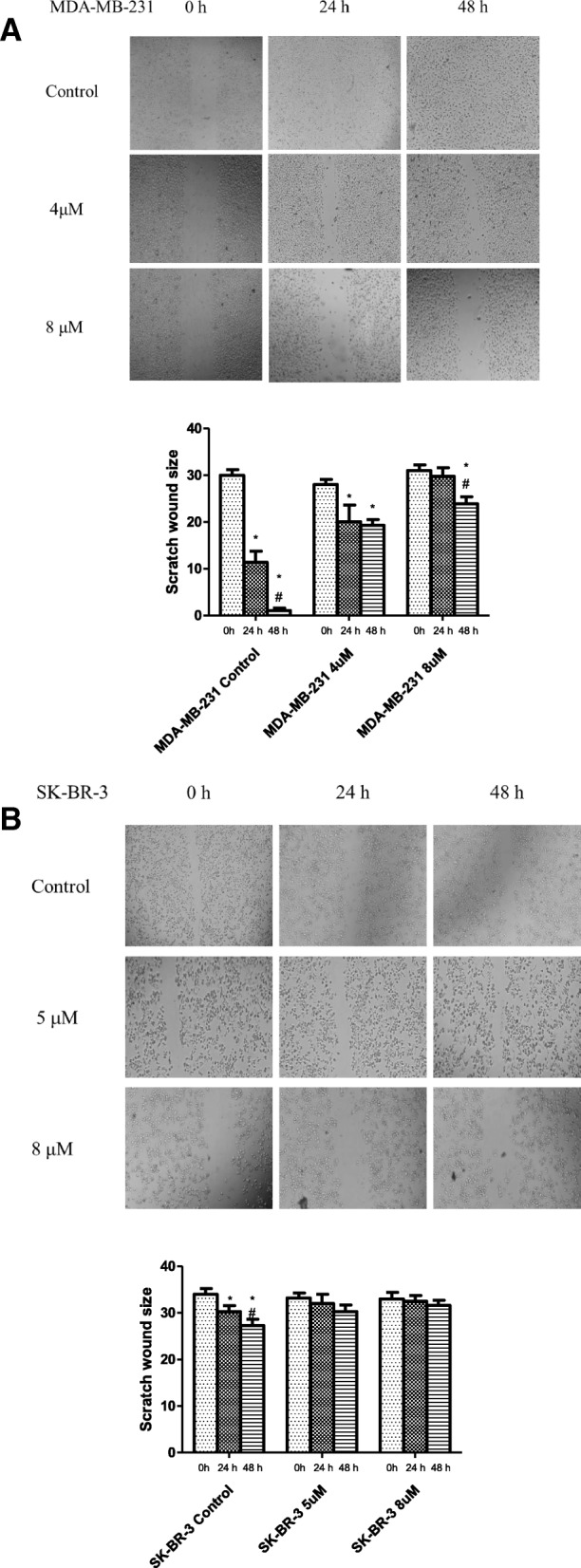
**a**, **b** Effect of eugenol on cell migration of MDA-MB-231 (**a**) and SK-BR-3 (**b**). Eugenol delayed the wound-healing time in human MDA-MB-231 (**a**) and SK-BR-3 (**b**) cells. The inhibitory effects of eugenol on the cells were determined using a wound healing assay. The distance of the scratch was measured in the control and eugenol groups using Image J software. Data are presented as mean ± SD (*n* = 3). * and # indicate a significant change from 0 and 24 h of incubation with eugenol respectively, at *p* < 0.05 using ANOVA followed by Tukey–Kramer post-ANOVA test

Wound healing assay was performed using eugenol treated MDA-MB-231 (4 and 8 μM) and SK-BR-3 (5 and 10 μM) cells to investigate the effect of eugenol on cell migration. Figure 3 shows that incubation with eugenol significantly reduced the migration ability of MDA-MB-231 and SK-BR-3 cells compared to untreated cells (*p* < 0.05). As shown in Fig. 3a, eugenol concentrations of 4 and 8 μM after 24 h delay MDA-MB-231 cell migration by 43 and 62%, respectively from 24 h control and, after 48 h delay MDA-MB-231 cell migration by 95 and 96%, respectively from 48 h control. Whereas eugenol concentrations of 5 and 8 μM after 48 h delay SK-BR-3 cell migration by 97% (Fig. 3b).

### Effect of eugenol on *MMP2* expression levels

**Fig. 4:**
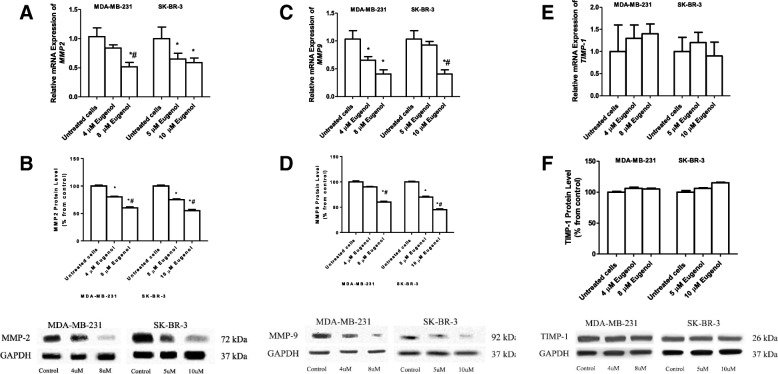
Effect of eugenol treatment on the mRNA expression of *MMP2* in MDA-MB-231 and SK-BR-3 (**a**), and on MMP2 protein levels (**b**), mRNA expression of *MMP9* in MDA-MB-231 and SK-BR-3 (**c**) and MMP 9 protein levels (**d**), mRNA expression of *TIMP-1* in MDA-MB-231 and SK-BR-3 (**e**), and TIMP-1 protein levels (**f**). Cells were treated for 48 h with eugenol (4 and 8 μM and 5 and 10 μM for MDA-MB-231 and SK-BR-3, respectively). Data are presented as mean ± SD (*n* = 3). * and # indicate a significant change from untreated, 4, and 5 μM eugenol respectively, at *p* < 0.05 using ANOVA followed by Tukey–Kramer post-ANOVA test

To determine the ability of eugenol to regulate the expression levels of genes involved in tumor metastasis, MDA-MB-231, and SK-BR-3 cells were incubated with two concentrations of eugenol, 4 and 8 μM, and 5 and 10 μM, respectively for 48 h. Subsequently, mRNA and protein levels were determined by real-time PCR and western blot. Figure 4a shows that incubation of MDA-MB-231 with 4 μM eugenol decreases the expression *MMP-2* by 1.25-fold whereas 8 μM significantly decreases it by 2-fold compared to both untreated and 4 μM treated cells. Similarly, SK-BR-3 treated with 5 and 10 μM eugenol shows a significant decrease in *MMP-2* expression levels by 1.6- and 1.72-fold, respectively, compared to untreated cells. To investigate whether the changes in *MMP2* mRNA levels are associated with alteration of protein levels, MDA-MB-231 and SK-BR-3 cells were incubated for 48 h with eugenol at 4 and 8 μM, and 5 and 10 μM, respectively. MMP-2 protein levels were then determined by western blot analysis. Similar to mRNA results, Fig. 4b shows that eugenol significantly decreases the protein levels of MMP2 in MDA-MB-231 by approximately 1.4- and 1.8-fold, and in SK-BR-3 by 1.75- and 2.1-fold, respectively compared to untreated cells, after normalization to GAPDH levels.

### Effect of eugenol on *MMP-9* expression levels

Figure 4c shows that eugenol concentrations of 4 and 8 μM significantly decrease the expression levels of *MMP-9* in MDA-MB-231 by 1.5- and 2.5-fold, respectively, compared to untreated cells. Incubation of SK-BR-3 with 5 μM eugenol causes an insignificant decrease in *MMP-9* expression whereas a high concentration (10 μM) significantly decreases its expression compared to untreated cells **(**Fig. 4c). Similar to the mRNA results, Fig. 4d shows that eugenol significantly decreases the protein levels of MMP-9 in MDA-MB-231 by approximately 1.5- and 1.7-fold, and in SK-BR-3 by 1.65- and 1.8-fold, respectively compared to untreated cells, after normalization to GAPDH levels.

### Effect of eugenol on TIMP-1 expression levels

Figure 4e shows the effect of different concentrations of eugenol (4 and 8 μM) on the expression levels of *TIMP-1* in MDA-MB-231 and (5 and 10 μM) in SK-BR-3 cells. Incubation of MDA-MB-231 with different concentrations of eugenol did not significantly alter the levels of TIMP-1. Likewise, incubation of SK-BR-3 with 5 and 10 μM eugenol increased the expression levels of TIMP-1 compared to untreated cells, albeit insignificantly (Fig. 4e). Similarly, eugenol treatment to MDA-MB-231 and SK-BR-3 resulted in insignificant changes in TIMP-1 protein levels in both cells (Fig. 4f).

### Effect of eugenol treatment on the expression levels of apoptotic genes

**Fig. 5:**
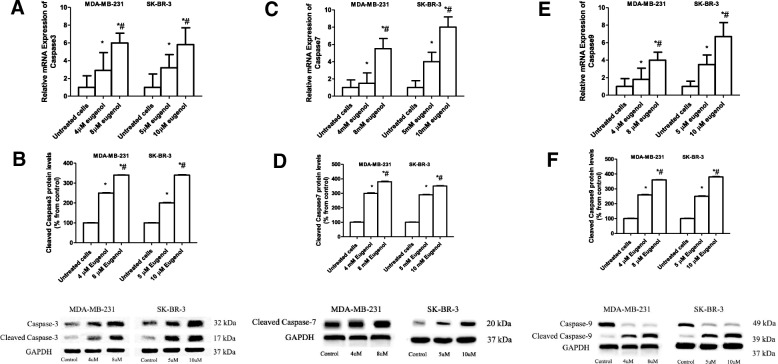
Effect of eugenol treatment on the expression levels of caspases in MDA-MB-231 and SK-BR-3 cell lines. MDA-MB-231 and SK-BR-3 cells were treated for 48 h with eugenol (4 and 8 μM, and 5 and 10 μM, respectively). The mRNA levels of *caspase-3* (**a**), *caspase-7* (**c**), and *caspase-9* (**e**) genes were quantified by RT-PCR and normalized to *GAPDH*. Protein expression levels of total and cleaved caspase-3 (**b**), cleaved caspase-7 (**d**) and total and cleaved caspase-9 (**f**) were determined by western blotting. Data are presented as mean ± SD (*n* = 3). * and # indicate a significant change from untreated, 4, and 5 μM eugenol respectively, at *p* < 0.05 using ANOVA followed by Tukey–Kramer post-ANOVA test

To determine the ability of eugenol to modulate the expression levels of caspase-3,-7, and − 9, MDA-MB-231 and SK-BR-3 cells were incubated for 48 h with increasing concentrations of eugenol (4 and 8 μM, and 5 and 10 μM, respectively). Subsequently, mRNA expression levels of apoptotic genes were quantified by real-time PCR. Figure 5 (a, c, e) shows that cells incubation with eugenol for 48 h significantly increased the mRNA expression levels of *caspase-3, − 7*, *and − 9* in MDA-MB-231 and SK-BR-3 in a concentration-dependent manner. In MDA-MB-231, the maximum induction levels of *caspase-3* (6.3-fold), *caspase-7* (5.5-fold), and *caspase-9* (4-fold) were observed at the highest concentration of eugenol tested, i.e.*,* 8 μM. Similarly, in SK-BR-3, the maximum induction levels of *caspase-3* (5.8-fold), caspase-*7* (8-fold), and *caspase-9* (6.7-fold) were observed at the highest concentration tested, i.e.*,* 10 μM. To confirm the effect of eugenol on the expression of apoptotic genes, breast cancer cell lines (MDA-MB-231 and SK-BR-3) were treated with different concentrations of eugenol and harvested after 48 h. Whole cell extracts were used to evaluate the levels of total and cleaved caspase-3, cleaved caspase-7, and total and cleaved caspase-9 proteins using immunoblotting and specific antibodies. Figure 5b, f show that eugenol triggered the cleavage of caspase-3 and -9, respectively, leading to a significant elevation in their active forms. Eugenol significantly overexpressed cleaved caspase-7 protein expression level (Fig. 5d) confirming the induction of apoptosis by eugenol in both triple negative and HER2 positive breast cancer cells.

**Fig. 6:**
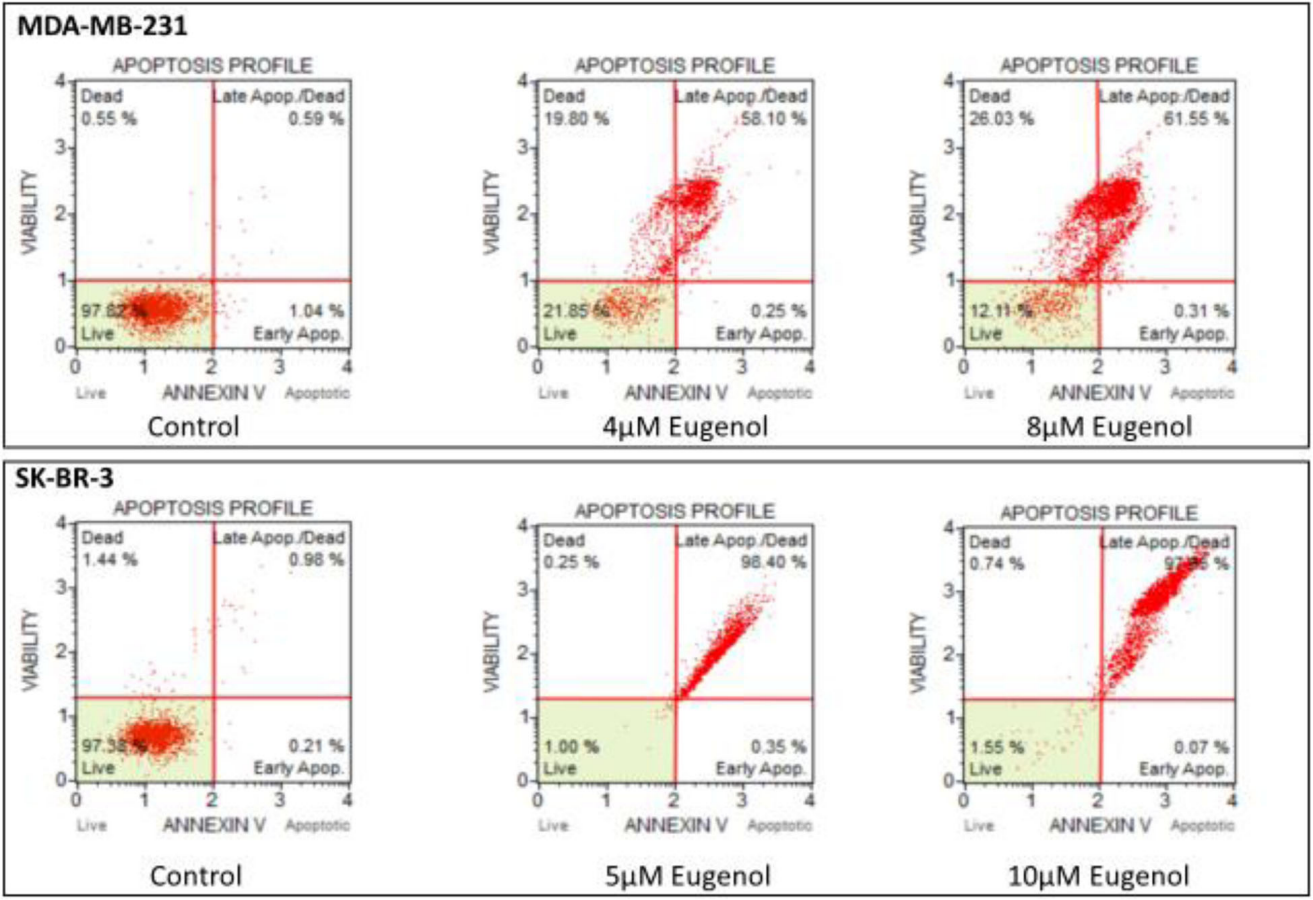
Effect of eugenol treatment on the percentage of apoptotic MDA-MB-231 and SK-BR-3 cells. Cells were treated for 48 h with eugenol (4 and 8 μM, and 5 and 10 μM, respectively). The percentage of cells undergoing apoptosis was determined using annexin-V staining. Cells were immediately analyzed on the Muse™ Cell Analyzer (Merck KGaA Co., Darmstadt, Germany)

To investigate the effect of eugenol concentrations on apoptotic genes and cell death, the percentage of apoptotic MDA-MB-231 and SK-BR-3 cells was determined by flow cytometry assay after 48 h of incubation with different doses of eugenol. Figure 6 shows that 97.82–97.38% of the untreated MDA-MB-231 and SK-BR-3 cells were healthy. However, MDA-MB-231 treated cells significantly increased the percentage of cells in late apoptosis from 0.59% (in untreated cells) to 58.10 and 61.55% and increased the percentage of dead cells from 0.55 to 19.8% and 26% in response to 4 and 8 μM of eugenol, respectively. Likewise, in SK-BR-3 cells, eugenol significantly increased the percentage of cells that experienced late apoptosis from 0.98% (untreated cells) to 98.40–97.65% in response to 5 and 10 μM eugenol respectively.

## Discussion

Eugenol is a member of the phenyl-propanoid category of compounds. It is used in medicine as a local antiseptic and anesthetic and in food products as a flavoring agent; however, it has some toxicity when used at high concentrations. The present study intended to investigate the possible anti-proliferative and apoptotic effects of eugenol against triple negative and HER2 positive breast cancer cell lines. Results of the proliferation assay indicated that eugenol had a concentration- and time-dependent effect on MDA-MB-231 and SK-BR-3 cell proliferation, suggesting the potential anti-proliferative and apoptotic effects of eugenol in MDA-MB-231 and SK-BR-3 cells. Eugenol at 40 and 60 μM markedly inhibited cell proliferation by more than 90% in both MDA-MB-231 and SK-BR-3 as determined by MTT assay. Another study suggested that 4 μM eugenol could inhibit cell proliferation in normal, ER-negative, and ER-positive breast cancer cells [22]. The cytotoxic effect of eugenol on G361 cells in the range of 0.5 to 2 mM was also reported. Moreover, the cell proliferation and migration of MCF7 ER + breast cancer cell line show dependence in time and eugenol dose [32]. The observed variation in the cytotoxic effect of eugenol across the literature could be due to the variation in used concentrations, purity of eugenol, and the source or types of cell lines.

In cancer, invasion, and metastasis, the primary cause of death, is mediated by a group of genes (e.g., MMPs) via degradation of the extracellular matrix with complicated steps [29, 33, 34]. Therefore, suppression of MMPs (expression or secretion) may be an effective strategy in preventing cell migration and invasion. Earlier trials with MMPs inhibitors in breast cancer revealed serious dose-limiting toxicity or failure to reach therapeutic plasma levels, which may be due in part, to their inability to target specific MMPs.

Multiple signaling pathways regulate the expression of MMPs [35, 36]. Thus, targeting genes in these signaling pathways may suppress or decrease metastasis and consequently reduce cancer mortality. The MMP-2 and -9 are collagenases involved in tumor invasion and metastasis through the degradation of collagen IV [37]. A correlation has been reported between a higher rate of distant metastases and high expression levels of MMP-2 and -9 [38]. Tissue inhibitor of metalloproteinases-1 has been suggested as a marker of prognosis and response to treatment in breast cancer. In the present study, MMP-2 and -9 mRNA and protein expression levels were significantly suppressed in cells after eugenol treatment, even though TIMP-1 was not changed. Further, eugenol treatment reduced the invasion and migration of breast cancer cells as shown in the wound healing assay. To the best of our knowledge, this is the first study to describe the anti-metastatic effect of eugenol against MDA-MB-231 and SK-BR-3 breast cancer cell lines. The observed efficient reduction of MMP gene expression levels during eugenol treatment suggested that eugenol can suppress triple negative as well as HER2-positive breast cancer metastasis. A study found that 100 μM eugenol has an antioxidant effect on human fibrosarcoma cells, and can inhibit MMP-9 activity and expression [39]. Another study found a correlation between high expression levels of MMP-2 and MMP-9 and lymph node metastasis and tumor staging in breast cancer patients [40]. Low expression levels of MMP-2 and MMP-9 in breast cancer patients may indicate a relatively good patient prognosis.

Eugenol is a cytotoxic compound and can trigger caspase-mediated cell death in breast cancer cells. Caspases have affected the mitochondria and upstream events of intrinsic apoptosis. Caspase-3, − 7, and − 9 have distinct roles during apoptosis with a possible feedback loop in the mitochondria. In the present study, eugenol significantly induced late apoptosis in MDA-MB-231 and SK-BR-3 in a dose-depended manner. It upregulated caspase-3, − 7, and − 9 expression levels in both MDA-MB-231 and SK-BR-3 along with a proportional increase in apoptosis. A previous study showed that eugenol suppressed E2F1/survivin and triggered apoptosis in breast cancer cells [22]. Eugenol may cause DNA degradation or cracking in MDA-MB-231 and SK-BR-3 cells and provoke a robust cytotoxic response [41]. Another study found that eugenol induces apoptosis in human osteosarcoma cells via activating caspase-3 and may play an important role in antitumor activity [42]. Cells deficient in caspase-3 or − 7 showed a delay in mitochondrial events of intrinsic apoptosis while Caspase-9 uncouples the mitochondria and increases ROS production [43]. A recent study found that eugenol enhances Bax, which may increase the release of cytochrome C from mitochondria and activate the caspase pathway needed for apoptosis [44].

## Conclusion

The present study demonstrates that eugenol exhibits an anti-breast cancer effect via targeting the caspase pathway and may be considered as a potential therapeutic agent for both triple negative and HER2-positive breast cancer.

## Abbreviations

cDNA: Complementary DNA
DMSO: Dimethyl sulfoxide
ECM: Extracellular Matrix
ELISA: enzyme-linked immunosorbent assay
ER: Estrogen Receptor
FDA: Food and Drug Administration
HER2: Human epidermal growth factor receptor 2
MMP2: Matrix metalloproteinases 2
MMP9: Matrix metalloproteinases 9
MMPs: Matrix metalloproteinases
MTT: 3-(4,5-dimethylthiazol-2-yl)-2,5-diphenyl tetrazolium bromide
PR: Progesterone Receptor
qRT-PCR: Quantitative Real-time polymerase chain reaction
RNA: Ribonucleic acid
TIMP-1: Tissue inhibitor metalloproteinases
TIMP-2: Tissue inhibitor metalloproteinases
TIMP-3: Tissue inhibitor metalloproteinases
TNBC: Triple-negative breast cancer
